# Caveolin-1 accumulation in the tongue cancer tumor microenvironment is significantly associated with poor prognosis: an *in-vivo* and *in-vitro* study

**DOI:** 10.1186/s12885-015-1030-6

**Published:** 2015-01-30

**Authors:** Marilena Vered, Meri Lehtonen, Lari Hotakainen, Emma Pirilä, Susanna Teppo, Pia Nyberg, Raija Sormunen, Ayelet Zlotogorski-Hurvitz, Tuula Salo, Dan Dayan

**Affiliations:** 1 Department of Oral Pathology and Oral Medicine, School of Dental Medicine, Tel Aviv University, Tel Aviv, 69978 Israel; 2 Institute of Pathology, The Chaim Sheba Medical Center, Tel Hashomer, Israel; 3 Department of Diagnostics and Oral Medicine, Institute of Dentistry, University of Oulu, Oulu, Finland; 4 Oulu University Hospital, Oulu, Finland; 5 Biocenter Oulu, University of Oulu, Oulu, Finland; 6 Medical Research Center, Oulu, Finland; 7 Institute of Dentistry, University of Helsinki, Helsinki, Finland

**Keywords:** Tongue cancer, Caveolin-1, Survival, Myoma organotypic model, Tumor microenvironment, Cancer-associated fibroblasts, Exosomes, Epithelial-mesenchymal transition

## Abstract

**Background:**

Caveolin-1 (CAV1) may be upregulated by hypoxia and acts in a tumor-dependent manner. We investigated CAV1 in tongue squamous cell carcinoma (TSCC) and its association with clinical outcomes, and studied *in vitro* possible ways for CAV1 accumulation in the tumor microenvironment (TME).

**Methods:**

TSCC cases (N = 64) were immunohistochemically stained for CAV1. Scores were separately assessed in the tumor and TME and plotted for association with recurrence and survival (univariate analysis with log-rank test). *In vitro* studies were performed on a 3D myoma organotypic model, a mimicker of TME. Prior to monoculturing HSC-3 tongue cancer cells, the model underwent modifications in oxygenation level (1%O_2_ hypoxia to upregulate CAV1) and/or in the amount of natural soluble factors [deleted by 14-day rinsing (rinsed myoma, RM), to allow only HSC-3-derived factors to act]. Controls included normoxia (21%O_2_) and naturally occurring soluble factors (intact myoma, IM). HSC-3 cells were also co-cultured with CaDEC12 cells (fibroblasts exposed to human tongue cancer). CAV1 expression and cellular distribution were examined in different cellular components in hypoxic and rinsed myoma assays. Twist served as a marker for the process of epithelial-mesenchymal transition (EMT). Exosomes isolated from HSC-3 media were investigated for containing CAV1.

**Results:**

Expression of CAV1 in TSCC had a higher score in TME than in the tumor cells and a negative impact on recurrence (p = 0.01) and survival (p = 0.003). Monocultures of HSC-3 revealed expression of CAV1 mainly in the TME-like myoma assay, similar to TSCC. CAV1+, alpha-smooth muscle actin (αSMA) + and Twist + CAF-like cells were observed surrounding the invading HSC-3, possibly reflecting EMT. RM findings were similar to IM, inferring action of HSC-3 derived factors, and no differences were seen when hypoxia was induced. HSC-3-CaDEC12 co-cultures revealed CAV1+, αSMA+ and cytokeratin-negative CAF-like cells, raising the possibility of CaDEC12 cells gaining a CAF phenotype. HSC-3-derived exosomes were loaded with CAV1.

**Conclusions:**

Accumulation of CAV1-TME in TSCC had a negative prognostic value. *In vitro* studies showed the presence of CAV1 in cancer cells undergoing EMT and in fibroblasts undergoing trans-differentiation to CAFs. CAV1 delivery to the TME involved cancer cell-derived exosomes.

## Background

A growing body of evidence has emerged in the last decade supporting the important role of the tumor microenvironment (TME) and its components in oral cancer growth, invasion and spread, especially in tongue cancer, the most frequent and aggressive type of oral cancer [1,2]. These biological features are most likely influenced by the molecular crosstalk between the cancer cells and various TME components, such as cancer-associated fibroblasts (CAFs) [3], inflammatory cells [3,4] and mesenchymal stem cells [5], hypoxic conditions, angiogenesis and extracellular matrix modulation. One of the factors involved in the molecular crosstalk are the exosomes, which are nano-vesicles secreted by the cancer cells [6,7]. Exosomes, which are now considered as one of the major intercellular communication vehicles, are able to transfer proteins and genetic material to other cells and to the extracellular matrix [8,9]. We have recently shown *in vitro* the presence of exosomal markers in both oral tongue cancer cells and within TME components [3].

Caveolin-1 (CAV1) is expressed in most cell types [10] and is present in a variety of cellular and extracellular compartments, thus explaining the variability of its functions and multiple interactions with signaling proteins that shape the outcome of its actions [11-14]. CAV1 has a role in both normal tissue homeostasis and pathological conditions, where it has been shown in some studies to be upregulated by the hypoxia-inducible factor (HIF)-α [13,14]. A crisis in oxygen availability or a tumor exhibiting a hypoxic signature leads to HIF-α–dependent up-regulation of CAV1 that enhances the oncogenic potential of tumor cells by increasing the cell’s proliferative, migratory, and invasive capacities [14]. It has recently been shown that the stroma of several human carcinomas, such as breast, colorectal and kidney, as well as that of metastatic melanomas, is enriched in CAV1-expressing CAFs. Furthermore, CAV1 expression in the CAFs of breast cancer correlated with low survival [13].

Most studies on CAV1 in oral squamous cell carcinoma (OSCC) examined its expression in the process of carcinogenesis. Clinico-pathological studies showed an increased immunoexpression of CAV1 in SCC tissue when compared to normal mucosa and precancerous (dysplastic) lesions [15]. Furthermore, quantum-dot immunohistochemistry in tissue microarrays showed an increased expression of CAV1 in stepwise carcinogenesis, from normal tongue mucosa, through hyperplasia, through precancerous lesions, and finally to primary SCC [16]. In addition, genetic studies on cell and tissue cultures as well as on human samples showed an increase in CAV1 gene expression in malignant cells compared to normal cells [17,18]. The aim of our study was to investigate the differential expression of CAV1 in cancer cells and in the TME of tongue SCC (TSCC) and to determine possible associations with clinical outcome. We monocultured a cell line of TSCC cells, HSC-3, on the 3D myoma organotypic model, considered as a best mimicker of TME due to the facts that the composition and variability of the soluble factors, the presence of various extracellular matrix proteins and glycoproteins as well as an inbuilt hypoxic environment enable cultured cancer cells to better manifest their malignant potential compared with collagen organotypic cultures [2,19,20]. Since the myoma discs by themselves lacked expression of CAV1, the notable expression of CAV1 in the cultures could be linked to HSC-3 delivery of CAV1 concomitant with the development of hypoxic conditions. In addition, we co-cultured HSC-3 cells with normal human gingival fibroblasts and a cell line of tongue cancer-related fibroblasts (CaDEC12 cells) in an attempt to highlight the co-expression of CAV1and αSMA in spindle cells surrounding the tumor islands and to determine any possible involvement of CAV1 in both epithelial-mesenchymal transition and fibroblast-to-cancer-associated fibroblast (CAF) trans-differentiation processes. Finally, we performed studies on monolayers of HSC-3 with the aim of determining whether the delivery of CAV1 from the HSC-3 cells into the TME was mediated by exosomes.

## Methods

### Expression of CAV1 in tongue carcinoma patients and association with clinical outcome

The study was approved by the IRB of the Chaim Sheba Medical Center (SMC 8437–11), Tel Hashomer, Israel. Accordingly, patient records/information was anonymized and de-identified prior to analysis. The study included tongue cancer patients (*n* = 64) diagnosed and treated between 1990 and 2006. They were comprised of 31 females (mean age 65 ± 11.7 years) and 33 males (mean age 57.4 ± 17.9 years), and classified as being in early-stage (stages I and II, *n* = 15) and late-stage (stages III and IV, *n* = 49) disease. The detailed clinical data on treatment and follow-up have been presented in detail in our previous study [3]. The clinical outcomes were measured by time to locoregional recurrence (LR) at the primary tumor site/cervical metastases/both and by overall survival (OS) of patients who were alive and free of disease/with disease at the last follow-up visit. The survival analysis included only patients followed-up for ≥18 months (*n* = 54).

Sections were prepared from paraffin blocks and immunostained with CAV1 (E294, Abcam, Cambridge, UK) according to the manufacturer’s instructions. Cytoplasmic expression of CAV1 was observed in the TME components (i.e., endothelial cells, CAFs, inflammatory cells). Since it was not possible to separate those components, the CAV1 expression in the TME was collectively termed “CAV-TME”. CAV1 expression in the tumor cells was termed “CAV-SCC”. The scores for CAV-TME and CAV-SCC in each case were calculated in a similar manner by considering the intensity of the staining (0 = no expression, 1 = weak expression, 2 = moderate expression, and 3 = strong expression) multiplied by the % of positive cells, with a maximum score of 3. In addition, the total CAV score in each case was determined as the sum of the CAV-TME plus the CAV-SCC scores. For statistical purposes, all the scores (i.e., TME, SCC and total) were classified as being low (score ≤ median) or high (score > median). Specimens of human placenta were used as positive control, and elimination of the primary antibody served as the negative control.

## Data analysis

For the CAV-stained patient tongue samples, univariate analysis was performed using the Kaplan–Meier method, and significance was confirmed by the log-rank test (SPSS, version 15, SPSS Inc., Chicago, IL, USA). Significance was set at *P* < 0.05.

### Tongue cancer cells – *In vitro* studies

#### HSC-3 cells mono- and Co-cultures using the 3D organotypic myoma model

This study was approved by the Ethics Committee of the Oulu University Hospital, Oulu, Finland.

The 3D organotypic myoma model was based on uterine leiomyoma tissue that was obtained from routine surgical operations after informed consent of the donors. The model was previously described in detail [19]. Homogeneous areas of the myoma served as sources for preparing discs for the organotypic culture by first cutting 3-mm slices with a disposable scalpel and further into discs with an 8-mm biopsy punch (Kai Industries Co., Gifu, Japan). The myoma discs were stored at −70°C in media with 10% DMSO (Sigma Aldrich). For preparing myoma-based organotypic cultures, myoma discs were equilibrated in media at room temperature (RT) for 1 hour. The myoma discs were placed into Transwell inserts (diameter, 6.5 mm; Corning, Inc., Corning, NY). For preparing mono-cultures, 3–4 × 10^5^ cells (e.g., oral tongue SCC HSC-3 cells, Japanese Collection of Research Bioresources Cell Bank, Japan) were cultured on the top of myoma tissue and allowed to attach overnight, and the myoma discs were removed from the Transwell inserts and transferred onto uncoated nylon discs resting on curved steel grids (3 × 12 × 15 mm) in 12-well plates with sufficient volume of media (1 ml). The myoma organotypic cultures were maintained for 14 days, and the media were changed according to the collagen organotypic culture protocol. In the co-culture assays, 2–3 × 10^5^ cancer cells were first mixed with 4 × 10^5^ other cell types (e.g., fibroblasts) and then cultured on myoma tissue and further processed as the mono-cultures [3,19].

In addition, rinsed myoma (RM) discs were prepared by rinsing discs in DMEM (Sigma Aldrich, Ayrshire, UK), gently rotating them at 4°C for two weeks and changing the media every three days [20]. The culturing procedures on the RM discs were identical to the above-described methodology.

Experiments were performed in normoxic and two types of hypoxic conditions: (a) 1% O_2_ hypoxia created by exposure of the assays in a hypoxic chamber (InVivo2 400, Ruskinn Technology, UK) for 20 h, and (b) overnight exposure to 100 mM cobalt chloride (CoCl_2_), a known hypoxia-mimicker, according to Brusewold et al. [21] and detailed elsewhere [20]. More specifically, HSC-3 monocultures were performed on both intact myoma (IM) and RM discs in both normoxic and hypoxic conditions. Co-cultures were performed on IM discs in normoxia.

##### Determining CAV1 expression in IM and RM discs

The IM and RM discs were snap-frozen in liquid nitrogen, crushed to a fine powder, dissolved in elution buffer overnight, and centrifuged (13,000 × g, 10 min), and the supernatant was collected and further prepared for Western blotting [20]. Briefly, the supernatant was re-suspended in 1x RIPA buffer, followed by Laemmli buffer. Proteins (14–20 μg/well) were separated by electrophoresis with 12% sodium dodecyl sulphate-polyacrylamide gel electrophoresis (SDS-PAGE). Overnight incubation with monoclonal rabbit CAV-1 antibody (1:5,000, clone E249, Abcam, Cambride, UK) was followed by a 1-h incubation with biotinylated polyclonal swine anti-rabbit secondary antibody (1:1000, Dako, Glostrup, Denmark). Membranes were developed with Pierce ECL Western blotting substrate.

##### HSC-3 organotypic mono-cultures and immunohistochemical stains

The RM discs that had been mono-cultured with HSC-3 (3–4 × 10^5^ cells) at 37°C with 5% CO_2_ for 14 days [3,20] were studied for the immunohistochemical expression of CAV1 and αSMA (serial sections). The same mono-cultures were also performed on the IM followed by the same immunostaining. RM/IM discs were fixed in 4% neutral-buffered formalin and embedded in paraffin [3,20]. Five micron-thick sections were immunostained with anti-caveolin-1 (E294, rabbit monoclonal, Abcam, Cambridge, UK, 1:250) or with anti-αSMA (1A4, mouse monoclonal, Dako, Glostrup, Denmark, 1:1000). Staining was demonstrated by a Dako REAL EnVision kit (peroxidase/DAB+, Dako) for mouse and rabbit primary antibodies. The above-described procedures on RM and IM HSC-3 mono-cultures were performed in both normoxic and hypoxic (1%O_2_) conditions [20].

IM and RM HSC-3 mono-cultures in normoxic and hypoxic conditions were triple immunostained with CAV1 antibody (1:100), pan-cytokeratin (1:50) and Twist (1:250; ab50581, Abcam, Cambridge, UK). A mixture of equal volumes of CAV1 and cytokeratin antibodies was incubated overnight at 4°C, rinsed and added to double-stain polymer adjusted for both mouse (HRP) and rabbit (AP) antibodies (Innovex Biosciences, Pinoles, CA, USA) for 30 min at RT. Sections were then stained with DAB for visualizing cytokeratin colored brown and with fast red (Permanent AP red kit, Zytomed, Berlin, Germany) for visualizing CAV1 colored purple. Antigen retrieval for Twist was performed by heating the slides in sterile water at 92° for 10 min and cooling for 20 min, followed by exposure to the Twist antibody at 4°C overnight and thereafter conjugation with rabbit/mouse polymer (Super-picture HRP, Invitrogen, Frederick, MD, USA) for 30 min at RT. Twist was finally visualized colored green (Permanent HRP green kit, Zytomed, Berlin, Germany).

The other series of triple immunostaining comprised cytokeratin (colored brown), Twist (colored purple) and alpha-smooth muscle actin (αSMA, colored green; 1:50; 1A4, Dako, Glostrup, Denmark). The procedure started with the mixing of equal volumes of Twist and cytokeratin antibodies followed by αSMA.

Assessment of the triple-immunostained sections was only qualitative and intended to demonstrate the localization of the staining among the cell types. Since the triple immunostaining revealed a similar pattern of expression of CAV1 in both RM and IM assays in normoxic and hypoxic conditions, the co-culture studies were performed only on the IM discs in normoxic conditions.

##### Co-cultures of HSC-3 with gingival fibroblasts and CaDEC12 cells on top of myoma discs

HSC-3 cells were co-cultured with human gingival fibroblasts (GFs, obtained from biopsies of healthy gingiva) [19] or with CaDEC12 cells (supplied by DEC) [3,5]. The latter are fibroblasts isolated from the connective tissue near tongue carcinoma. These cells were characterized at passage 3 for the expression of lineage-specific markers by flow cytometry and immunohistochemistry. They expressed the fibroblast markers vimentin (98.67% positive cells) and platelet-derived growth factor receptor (PDGFR)-B (97.60% cells), and were negative for epithelial (epidermal surface antigen [ESA] 0.09% positive cells), endothelial (CD31 0.65% positive cells), and hematopoietic (CD45 0.25% positive cells) lineage-specific markers. They were also negative for αSMA. Very low levels of the pericyte/mesenchymal stem cell marker CD146 (1.48% positive cells) were detected. In addition, p53 (exons 5–9) mutation analysis was performed using genomic DNA, and it revealed that CaDEC12 fibroblastic cells were harboring wt p53, while the epithelial cells that had been derived from the same tumor showed a p53 mutation in exon 7 (codon 772 G > A) [3,20].

In the co-culture assays, 2–3 × 10^5^ HSC-3 cells were first mixed with 4 × 10^5^ GFs or CaDEC12 cells and then cultured on myoma discs [3]. The immunostaining procedures and the assessment of CAV1and αSMA in the co-culture assays were done as described above. In addition, sections from the co-cultures were submitted to double immunostaining with CAV1, colored brown, and pan-cytokeratin (AE1/AE3, Dako, 1:200, 30 min RT), colored grey, using the SG color chart (Vector Laboratories, Burlingame, CA, USA). Furthermore, some of the co-cultures were stained with αSMA, as described above.

Assessment of CAV1 expression in the co-cultures was performed semi-quantitatively on a scale of 0 to 4: 0 = no staining, 1 = weak staining in <50% cells, 2 = weak but extensive (>50% cells) staining, 3 = strong staining in <50% cells, and 4 = strong staining in >50% cells, as previously described [3]. The HSC-3 and the cells immediately surrounding or adjacent to the tumor islands/clusters were assessed separately for the upper region of the section (the area of the lining tumor cells and the adjacent sub-tumoral stroma) and in the lower region (the area of invading tumor islands and the surrounding cells). This semi-quantitative assessment attempted to give a general overview of the staining patterns and, as such, was not further processed by statistical analysis. Sections of HSC-3 monocultures immunostained with CAV1 served as controls. Assessment of αSMA was qualitative and intended to show the presence and distribution of spindle-shaped, positively stained cells and their relations to the tumor islands/clusters.

### HSC-3 monolayer cultures

Similarly to the myoma assays, HSC-3 monolayers were cultured in normoxic and in hypoxic (1% O_2_) and hypoxia-mimicking (CoCl_2_) conditions.

#### CAV1 expression in HSC-3 cell extracts

Cell extracts were processed for Western blotting, as described above. The same procedure for CAV1 blotting was performed on cell media after centrifugation at 3000 × g for 15 min to remove cell debris.

#### HSC-3 cell-media exosome isolation

HSC-3 monolayers were grown in normoxia in DMEM/F12-medium supplied with 10% fetal bovine serum (both from Life Technologies, Carlsbad, CA, USA), 50 μg/ml ascorbic acid, 0.4 μg/ml hydrocortisone, 100 U/ml penicillin, 100 μg/ml streptomycin and 250 ng/ml Fungizone (all from Sigma Aldrich) until 90% confluence. The cells were then washed with PBS, and incubation continued in Opti-MEM® I Reduced Serum Medium (Life Technologies) for 24 h. One bottle of 15 × 10^6^ HSC-3 cells was similarly treated and incubated with Opti-MEM with the addition of 50 μM 4-aminophenylmercuric acetate (APMA, Sigma Aldrich, St. Louis, MO, USA) to induce exosome release, as shown previously [22]. Incubation lasted for 6 h, after which the media were collected and the exosomes were isolated with ExoQuick-TC™ kit (Systems Biosciences, CA, USA) according to the manufacturer’s instructions. Briefly, the medium was centrifuged at 3000 × g for 15 min to remove cell debris. ExoQuick-TC solution was added to the supernatant in a 5:1 ratio and kept at +4°C overnight. The solution was then centrifuged 1500 × g for 30 min and the supernatant was removed. The precipitate was suspended in 100–200 μl of PBS for ELISA and immuno-electron microscopy (immunoEM) or 1 × RIPA-buffer for Western blotting.

##### ELISA of the Exoquick-isolated exosomes

ELISA analyses were performed for the identification of the exosomal markers, CD63, CD9 and CD81 using the ExoELISA™ kits (System BioSciences, CA, USA) according to the manufacturer’s instructions. After overnight incubation at 37°C, CD63, CD9 or CD81 antibodies were added to the wells (one antibody/well) and incubated for 1 h at RT, followed by horseradish peroxidase-linked secondary antibody for 1 h at RT. TMB ELISA substrate was added, incubated for 30 min at RT and stop solution was added to end the reaction. The amount of proteins was determined by reading the optical density using a Victor 3 microplate reader (Perkin Elmer, MA, USA) at 450 nm. The amount of exosomal proteins was plotted against the standard curves.

ELISA for determining CAV1 was performed using a human CAV1 ELISA kit (MyBioSource, Inc., CA, USA) according to the manufacturer’s instructions. Briefly, 50 μl of the PBS-suspended exosomal precipitates or CAV1 standards were added to the antibody pre-coated microtiter plate. Five μl of balance solution was added to each well followed by 100 μl of conjugate. Incubation continued for 1 h at 37°C. Substrates A and B were added to each well, and incubation continued for 15 min at 37°C. Stop solution was used to end the reaction, and optical density was determined at 450 nm.

##### Western blotting of CAV1 in Exoquick-isolated exosomes

Laemmli buffer with 2-mercaptoethanol was added to the exosome pellets, followed by incubation in boiling water. The subsequent procedures were the same as those detailed above.

##### ImmunoEM of Exoquick-isolated exosomes

The precipitates were laid on Formvar carbon-coated, glow-discharged grids for the immunoEM of the Exoquick-isolated exosomes. The grids were first incubated in a blocking serum containing 1% BSA in PBS. Antibodies and gold conjugates were diluted in 1% BSA in PBS. The grids were then incubated with mouse CD63 primary antibody (Abcam, Cambridge, UK) or with CAV1 (Abcam, Cambridge, UK) for 20 min, followed by rabbit anti-mouse IgG-gold with a particle size of 10 nm (Aurion, Wageningen, The Netherlands) for 20 min. The controls were prepared by replacing the primary antibody with PBS. The grids were stained with neutral uranyl acetate and embedded in methylcellulose/uranyl acetate and examined with a Tecnai Spirit transmission electron microscope (FEI, Eindhoven, The Netherlands). Images were captured by a Quemesa CCD camera (Olympus Soft Imaging Solutions GMBH, Munster, Germany).

## Results

### Tongue cancer patients

**Figure 1:**
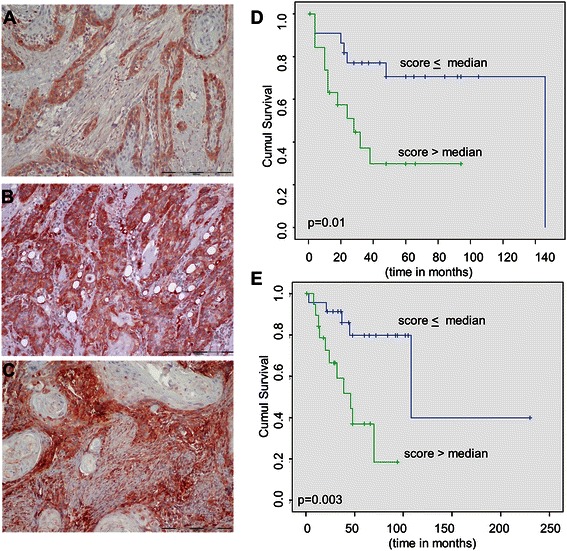
**Caveolin-1 immunostaining patterns in human tongue sections and association with clinical outcomes. A**. Staining in the tumor is principally limited to the peripheral cells and is assessed with a score of 1.0 (50% for extent of staining and 2 for staining intensity). In contrast, there is almost no staining of the tumor microenvironment (TME) that was given a score of 0.1 (10% for extent of staining and 1 for staining intensity). **B**. Most of the tumor cells are strongly positive (a score of 3) and TME is negative (a score of 0). **C**. Caveolin-1 is moderately positive in the tumor cells (score of 0.9), but it predominates in the TME (score of 3) (**A**, **B**, **C** - bar = 200 μ). **D**. Kaplan-Meyer analysis shows a significantly negative influence of high CAV-TME score on locoregional recurrence (p = 0.01). **E**. Kaplan-Meier analysis shows a significantly negative influence of high CAV-TME score on overall survival (p = 0.003).

The present study is the first to investigate the accumulation of CAV1 in cancer cells and TME components in a series of tongue cancer cases and its association with clinical outcome. CAV-SCC was positive in all 64 cases (median score 0.9, range 0.1–3), with only 11 cases being greater than the median and only one case with a score of 3. CAV-TME was positive in 54 cases (median 0.6, range 0–3), with 24 cases being greater than the median and 11 cases having a score of 3. CAV-total had a median of 1.6 (range 0.1–4.5). Figure 1A-C illustrates the CAV1 staining patterns. Univariate analysis demonstrated that only CAV-TME was associated with clinical outcome: a high score had a negative impact on both recurrence (*P* = 0.01) and survival (*P* = 0.003) (Figure 1D and E, respectively). Although we detected evidence of CAV1 in the tumor cells in all the examined cases, as reported in previous studies [15-18], we found no significant association with either recurrence or survival. These findings motivated us to perform a series of *in vitro* studies in which we attempted to determine the mechanisms of CAV1 accumulation within the TME.

As mentioned in “Methods”, section on tongue cancer patients, the present tumors were previously examined for abundance of CAFs and the profile of the inflammatory cells [3]. CAV-TME was found to be positively correlated with CAF density (r = 0.497, p < 0.001**)**.

### *In vitro* studies

#### Myoma organotypic model

##### Rinsed and intact myoma discs (RM and IM, respectively): CAV1 expression

**Figure 2:**
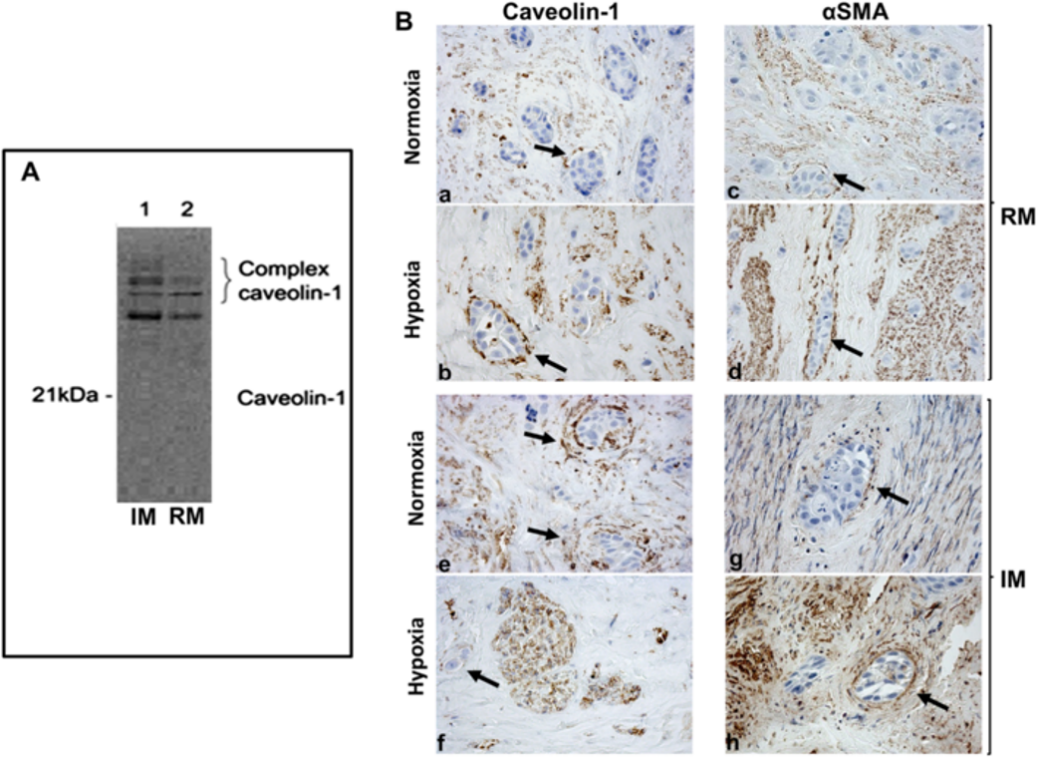
**Caveolin-1 expression in HSC-3 cell myoma monocultures. A**. Western blotting: caveolin-1 is detected as complex forms in intact myoma (IM) tissue disc extracts (without added cells), and, to a lesser degree, in the rinsed myoma (RM, without added cells and without soluble factors) disc extracts. Monomeric caveolin-1 is absent from both IM and RM tissue extracts. **B**. HSC-3 monocultures within rinsed myoma (RM) in both normoxic and hypoxic conditions **(a, b)** show delicate, spindle, caveolin-1-positive cells closely surrounding invading HSC-3 islands. Alpha-smooth muscle actin immunostaining in normoxic and hypoxic conditions **(c, d)** shows findings similar to caveolin-1 in terms of cell distribution and morphology. HSC-3 monocultures within intact myoma (IM) immunostained with caveolin-1 **(e, f)** and alpha-smooth muscle **(g, h)** show findings similar to those of the RM, respectively. CAV1 and alpha-smooth muscle actin positively stain the spindle cells surrounding the invading HSC-3 tumor islands (indicated by arrows). In all sections, the HSC-3 cells are only occasionally CAV1-positive in both RM and IM, and in both normoxia and hypoxia. However, the spindle cells are usually CAV1-positive, showing a high staining intensity.

Although cells within the myoma discs are no longer expected to be viable [19], the microenvironment is rich in soluble factors with necessary functions for facilitating tumor growth and invasion. Depletion of these factors (including CAV1) by rinsing the myoma discs approached a state in which factors released into the myoma assay originated from the cultured HSC-3 cells. Western blotting of either RM or IM discs showed no monomeric 21 kDa form of CAV1 but only complex forms of immunoreactants (Figure 2A). Thus, it can be inferred that CAV1 in the myoma model is expected to originate from the cell lines cultured in this assay.

##### HSC-3 monocultures in hypoxic and normoxic conditions: CAV1 and αSMA expression

Since expression of CAV1 is upregulated in hypoxic conditions [13,14], mono-cultures were submitted to both hypoxia and normoxia. We were interested in determining whether growing the HSC-3 cells on the myoma discs in different oxygenation conditions could have an effect on the intra- or extracellular distribution of CAV1. Although we found no CAV1 protein product in either the RM or IM discs, we were interested in exploring any possible differences in the pattern of expression of CAV1 between the two types of normoxic and hypoxic monocultures.

It was interesting to note that in all the monoculture assays, CAV1 was immunohistochemically identified mainly in the “TME” cells, represented by the myoma discs, and to a considerable lesser extent within the HSC-3 cells. In the normoxic RM monocultures (devoid of myoma-related mediators), CAV1-positive cells were present in delicate, spindle-shaped cells concentrically surrounding the periphery of the invading HSC-3 islands (Figure 2B). This pattern of CAV1 expression and cellular distribution was similar to that observed in the IM sections, therefore it can be assumed that evolvement of the spindle cells and their peri-tumoral organization were probably mediated through signals originating from the HSC-3 cells. In addition, since the same changes were seen in both normoxic and hypoxic conditions, it is proposed that hypoxia did not change the distribution of CAV1, given that the myoma model is characterized by an in-built potential for a hypoxic environment [20]. Furthermore, we performed αSMA immunostaining since the peri-tumoral location of the CAV1-positive spindle cells recapitulated the arrangement of CAFs in the human tongue SCC case. We found that αSMA-positive spindle-shaped cells were also present in RM and IM in both norrmoxic and hypoxic conditions in distributions that were almost identical to those of CAV1-positive cells (Figure 2B). Sections from CoCl_2_ hypoxia-mimicking conditions in RM and IM assays were similarly stained and yielded comparable results (data not shown).

In malignant epithelial cells, factors, such as peroxisome proliferator-activated receptor-χ, are responsible for an increased expression of CAV1 [23]. In contrast, we found that CAV1 expression was higher in the TME-like myoma than in HSC-3 cells in the monoculture assays, and we therefore inferred that CAV1 was exported from the HSC-3 cells into the TME and assumed that this was performed through the exosomal route, which will be further described in the monolayer assays.

##### HSC-3 monocultures in hypoxic and normoxic conditions: triple immunostains

**Figure 3:**
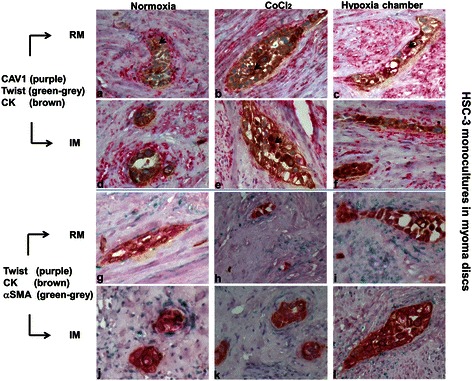
**HSC-3 monocultures with two types of triple immunohistochemical panels.** Caveolin-1 (CAV1), Twist and cytokeratin (CK) in rinsed and intact myoma in normoxia and hypoxia [cobalt chloride (CoCl_2_) or in hypoxia chamber with 1% O_2_)] **(a-f)** show similar patterns of expression in all settings: positive nuclear staining of the invading HSC-3 islands with Twist (most nuclei) and diffuse cytoplasmic staining with cytokeratin and, to a lesser extent, with CAV1 (arrows). In addition, delicate, spindle-shaped cells that closely surround these invading tumor islands in a concentric pattern are also CAV1-positive. CK and alpha-smooth muscle actin (αSMA) in rinsed and intact myoma in normoxia and hypoxia [cobalt chloride (CoCl_2_) or 1%O_2_)] **(g-l)** show similar patterns of expression in all settings: positive nuclear staining of the invading HSC-3 islands with Twist (most nuclei) and diffuse cytoplasmic staining with CK. In addition, delicate, spindle-shaped cells that closely surround these invading tumor islands in a concentric pattern are αSMA-positive.

Since in the previously described experiment (i.e., IM and RM HSC-3 monocultures in normoxic and hypoxic conditions) the CAV1-positive spindle-shaped cells were found in an intimate relation to the HSC-3 tumor clusters, we raised the possibility that the origin of these cells could be from HSC-3 cells that had undergone a process of epithelial-mesenchymal transition (EMT). Therefore, serial sections from that experiment were triple immunostained with CAV1, pan-cytokeratin and Twist, an EMT marker. We found that invading cytokeratin-positive HSC-3 cells in normoxic RM sections had a nuclear expression of Twist, and that a number of cells also showed cytoplasmic expression of CAV1 (Figure 3). The delicate spindle-shaped cells that were concentrically arranged in the closest periphery of the HSC-3 cells were CAV1-positive. This configuration of the spindle cells was identified exclusively at the periphery of invading HSC-3 cells and not in areas of myoma beyond this vicinity or in areas of myoma devoid of HSC-3 cells. These findings were similar to those of hypoxic RM sections, thus once more supporting the conclusions that a myoma constitutes a natural hypoxic environment and that factors secreted from the HSC-3 cells can have autocrine and/or paracrine effects. These findings are reflected by the evolvement of the spindle cells at the periphery of the HSC-3 tumor islands. Normoxic and hypoxic IM sections showed a similar set of results.

The other triple immunostaining series aimed to show that HSC-3 cells (cytokeratin-positive) may undergo EMT (Twist-positive) and be a source for CAF-like cells (αSMA+) (Figure 3). Invading cytokeratin-positive HSC-3 clusters of cells were usually also Twist-positive in normoxic RM sections. A part of these clusters was surrounded at their periphery by layers of fine cells with spindle-shaped morphology of an αSMA-positive phenotype. Similar findings were present in hypoxic RM sections and in normoxic and hypoxic IM sections, further highlighting that hypoxia did not have any effect on the myoma assay and that the described phenotypical changes did not depend on the presence of myoma-related mediators but rather were self-supplied by the HSC-3 cells.

Since the triple immunostainings revealed similar patterns of expression in both RM and IM assays in normoxic and hypoxic conditions, co-culture studies were performed only on the IM in normoxic conditions.

### Co-cultures

We used a cell line of fibroblasts obtained from the vicinity of a tongue SCC, i.e., CaDEC12 cells, because CAF cell lines are not yet available. Having been exposed to carcinoma cells and associated TME, these fibroblasts assumedly had undergone a “priming” process towards trans-differentiation into CAFs. This was reflected by the ubiquitous expression of PDGFR in the CaDEC12 cells, as PDGFR is one of the known markers that are expressed in the activation state of stromal fibroblasts of solid tumors while gaining a CAF phenotype [24].

**Figure 4:**
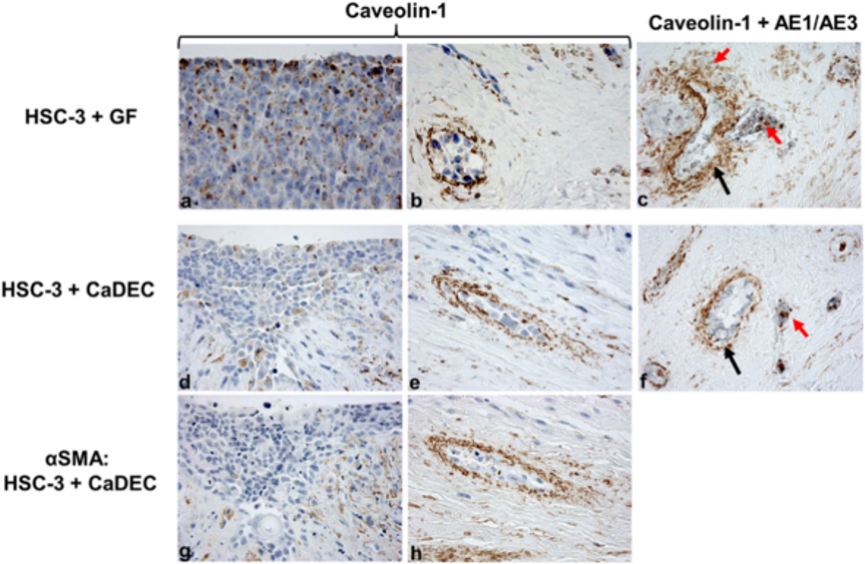
**HSC-3 co-cultures: intact myoma, normoxia.** HSC-3 cells were cocultured with gingival fibroblasts (GFs) or with fibroblasts isolated from a human tongue tumor (called CaDEC12 cells). A few caveolin-1-positive cells are seen in the upper region tumor cells **(a, d)**. Caveolin-1-positive cells are spindle-shaped and concentrically arranged around the invading tumor islands in the lower regions **(b, e)**. Serial sections of **d** & **e** (**g & h**, respectively) of the lower region also show peri-HSC-3 spindle cells positive for alpha-smooth muscle actin (αSMA). Double immunostaining with pan-cytokeratin (AE1/AE3, grey color) and caveolin-1 (brown color) in co-cultures of HSC-3 + GF **(c)** and HSC-3 + CaDEC12 **(f)** shows that the spindle-shaped cells surrounding the invading tumor islands are only caveolin-1-positive (black arrows). Some of the cells within the tumor islands are positive for both cytokeratin and caveolin-1 (**c**, **f**, red arrows).

CAV1 immunoreaction in the HSC-3-CaDEC12 cocultures was low (a score of 1) in the upper region of the myoma in the spindle-shaped, the peri-HSC-3 cells as well as in the HSC-3 cells. The peri-HSC-3 cells in the lower region of the myoma showed a high immunoexpression (a score of 4), whereas the HSC-3 cells demonstrated a low expression (a score of 1) (Figure 4, Table 1). These findings may suggest that a substantial part of the CAV1 is transported from the HSC-3 cells into the spindle cells in the lower region. The expression of CAV1 in the HSC-3-GF co-cultures seemed to be similar in both spindle cells and HSC-3 cells, irrespective of the myoma region. These findings differed from those of the HSC-3-CaDEC12 co-cultures and could be attributed to the different source of the fibroblasts and their crosstalk with the HSC-3 cells. The results of the control HSC-3 monocultures were similar to those of the HSC-3-CaDEC12 cocultures, raising the possibility that introduction of normal GFs could have an effect on the expression of CAV1 within the HSC-3 cells and on its distribution to the surrounding cells within the myoma. Collectively, we suggest that, similarly to other types of carcinomas (e.g., breast, kidney and colon) [13], CAV1 accumulates within the CAFs in tongue cancer as well.

**Table 1:** **Scores of caveolin-1 expression in normoxic intact myoma in mono- and co-cultures**

	Peri-HSC-3 spindle cells		HSC-3	
Mono- and co-cultures	Upper region^b^	Lower region^c^	Upper region^b^	Lower region^c^
Myoma + HSC-3 + CaDEC12	1	4	1	1
Myoma + HSC-3 + GF^a^	3	3	3	3
Myoma + HSC-3	1	4	1	1

^a^Gingival fibroblast; ^b^region immediately adjacent to the overlying HSC-3 cells; ^c^deep region within the myoma tissue containing invading HSC-3 islands/clusters.

αSMA-positively stained cells were seen in a peri-HSC-3 location, surrounding tumor islands/clusters in the lower region. Serial sections clearly demonstrated that spindle-shaped cells were positively stained for both CAV1 and αSMA.

Double immunostaining with pan-cytokeratin and CAV1 showed that the spindle-shaped cells surrounding clusters of invading tumor islands/clusters were positive for CAV1 and not for cytokeratin, thus highlighting the possibility that the GFs or CaDEC12 cells could serve as a source for the spindle-shaped cells. Cells within the invading tumor islands/clusters were occasionally positive for both cytokeratin and CAV1.

In line with the above-described co-localization of CAV1 and αSMA in CAF-like cells, it has been elegantly shown that CAV1 promotes αSMA expression in fibroblasts (i.e., CAFs) and that CAV1-positive CAFs are potentiated to exert force-dependent contraction of the surrounding matrix in a Rho/Rho kinase-dependent manner [13]. As a result, the matrix collagen fibers undergo remodeling by being aligned in a parallel manner, thus creating directional invasion tracks for the migrating tumor cells [13,25].

We performed an additional set of experiments on HSC-3 monolayers since we were interested in exploring the possibility that the autocrine and paracrine effects of CAV1 were, at least in part, carried out by its using the exosomal transport route.

### HSC-3 monolayers

This is the first report to show that tongue cancer cells use exosomes for transferring CAV1 to the TME and that these exosomes are apparently ingested by different types of cells within the TME, such as CAFs.

#### HSC-3 monolayers in hypoxic and normoxic conditions: CAV1 expression in cell extracts

**Figure 5:**
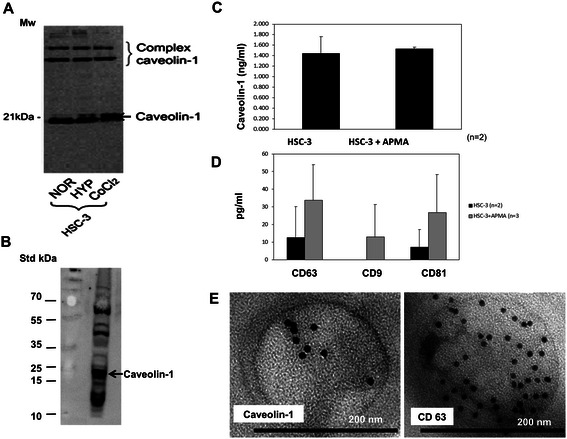
**Caveolin-1 in monolayer HSC-3 extracts (A) and in exosomes isolated from their media (B-D). A**. Caveolin-1 protein are seen as strong monomeric bands (~21 kDa) (and in complex forms, ~50 kDa – 60 kDa) in whole HSC-3 cell extracts cultured in monolayers under normoxic (NOR) conditions. Hypoxic (HYP) oxidative conditions and hypoxia mimicking CoCl_2_ media yield similar results. **B**. Exosomes isolated from the media of HSC-3 normoxic monolayer submitted to Western blotting for caveolin-1 show strong bands in the 20–23 kDa form (and in complex forms, ~50 kDa – 60 kDa). **C**. Results of ELISA tests performed on the exosomes isolated from HSC-3 monolayer media reveal caveolin-1 expression. The results are presented as the mean amount (±SD) of each protein of experiments with and without APMA induction. **D**. ELISA performed on HSC-3 exosomes shows the expression of the exosomal markers, CD63, CD9 and CD81. The results are presented as the mean amount (±SD) of each protein, with and without APMA induction. **E**. Immuno-electron microscopy that was performed on the monolayer media-isolated exosomes features round-shaped, membrane-bound nanoparticles that contain dots positive for both caveolin-1 and CD63.

First, we investigated CAV1 expression in HSC-3 cell extracts in normoxia as well as in hypoxic (1%O_2_) and hypoxia-mimicking (CoCl_2_) conditions, similarly to what had been investigated in the myoma model. Western blotting for CAV1 detected a corresponding 21 kDa band in normoxia and bands of a similar width in the hypoxic conditions. This highlights the ability of HSC-3 cells to express CAV1 and demonstrates that this ability was not influenced by hypoxia (Figure 5A). In addition, Western blotting for CAV1 performed on the media of the monolayer cells was negative (data not shown).

Since we found no difference in the CAV1 level among HSC-3 cell extracts from various oxidative conditions, we proceeded to the isolation of exosomes that had been performed from the media of only the normoxic monolayers.

#### HSC-3 monolayer media exosomes – CAV1

Similar to what we had shown in the cell extracts, Western blotting performed on Exoquick-isolated exosomes clearly showed a band of corresponding weight to that of CAV1 (Figure 5B).

##### HSC-3 Exosomes - ELISA and immunoEM

Exoquick-isolated exosomes studied by ELISA also demonstrated CAV1 expression in these exosomes (Figure 5C), in parallel to the presence of all three known exosomal markers, CD63, CD9 and CD81 [7] (Figure 5D). The expression of the examined proteins was generally enhanced by APMA, implying that the exosomes were restrained within the cytoplasmic compartment of the HSC-3 cells grown in monolayers in the absence of adequate stimuli. These findings differ from those of the HSC-3 cells that were monocultured on the myoma model, where most of the CAV1 expression was observed in the TME-like myoma rather than in the HSC-3 cells, suggesting that CAV1 accumulation within the TME was controlled by different cell-environment relationships. ImmunoEM nicely showed round-shaped, membrane-bound nanovesicles, ~150 nm in diameter, and positive for intra-vesicular CAV1 (Figure 5D). Furthermore, these nano-vesicles showed positivity to CD63, a classic exosomal marker (Figure 5E). Immunoreaction was not detected in the negative immunoEM controls (data not shown).

## Discussion

The contribution of CAV1 to cancer progression, whether expressed in the cancer cells or in the TME components, seems to be complex and controversial and apparently tumor-related as well. Studies on the expression of CAV1 in both cell lines and human samples of oral cancer were recently reviewed [26]. Some studies reported that the expression of CAV1 increased in a stepwise manner following carcinogenesis, while others claimed that it was inactivation of CAV1 that played a role in oral cancer. Expression of CAV1 in the TME of oral cancer has not been previously examined. We were the first to show that expression of CAV1 in human TSCC in the TME components was higher than in the tumor cells and that it had a negative impact on the clinical outcomes. Using the 3D myoma organotypic model for the *in vitro* studies had the advantage of a natural hypoxic environment. We found CAV1 expression to be higher in the TME-like myoma environment compared to the cultured cancer cells, similar to the TSCC samples, even when severe hypoxic conditions were further induced in the myoma assays.

Our clinical findings were not in agreement with those reported from breast and prostate cancer patients, where the absence of CAV1 in the TME was associated with advanced stage, recurrent disease, metastatic spread and poor survival [27,28]. Furthermore, findings from our *in vitro* myoma model contrasted those from investigations of other types of carcinoma cell lines that were cultured on traditional collagen assays, especially of gastric [29] and breast [30-32] origins. Those latter studies showed that breast cancer cells were able to induce oxidative stress (via a yet unknown mechanism) and/or hypoxic conditions (via HIF-α) in CAFs, which, in turn, activated autophagic and lysosomal degradation of CAV1 as well as of dysfunctional mitochondria. The resulting aerobic glycosylation in CAFs generated oxidative metabolic products, such as lactate, that were used as nutrients by the cancer cells. The oxidative stress within the CAFs was also associated with advanced genetic instability in the cancer cells and, hence, an increased biological aggressive behavior. According to this model, it would be expected that the presence of CAFs will be inversely related to the expression of CAV1. Contrarily, our clinical results showed a significant correlation between the presence of CAFs and CAV1, a finding which can not be explained by the CAV1 autophagy theory. Furthermore, our *in vitro* results showed concomitant expression of CAV1 in αSMA + −CAF-like cells. Similar to our results, Goetz et al., who studied breast carcinoma, concluded that CAV1 expression in the CAFs of breast cancer correlated with low survival, and that CAV1 expression in fibroblasts favored directional migration and invasiveness of carcinoma cells *in vitro* [13].

In addition to autophagy that delivers cytosolic components and organelles, extracellular material and plasma membrane proteins are delivered to the lysosomes via endocytosis and multivesicular bodies (MVBs) [7,33]. Alternatively, in order to alleviate cellular stress, MVBs are directed to release their content of unwanted/damaged materials into the extracellular environment as exosomes. It can be assumed that when CAV1 is “unwanted”, it is removed by the endocytic-MVB-exosomal route rather than by the autophagy pathway, by virtue of its membranous location. The balance between exosome release and autophagy induction is probably regulated by the cellular metabolic state, but this is still not fully understood. Several assays showed that cellular stress, such as thermal or oxidative stress, enhanced the release of exosomes [34]. Furthermore, it has been shown that exosomes that were released under oxidative stress provided recipient cells with resistance to oxidative stress and with improved survival [35].

## Conclusions

Specific exosomal transport of CAV1 has been shown in only a few systems thus far, and it included exosomes in the plasma of melanoma patients [36] and in the vesicular organelles (prostasomes) secreted by human prostate cancer cells [37]. One of the ways that can enable the accumulation of CAV1 in the TME is its being secreted by exosomes. As such, exosomal CAV1 can be present in the extracellular matrix or it may be ingested by cells that undergo EMT, by CAFs or by other types of cells. In the context of oral cancer, the role of CAV1 in the process of EMT and/or in fibroblast-to-CAF trans-differentiation should be elucidated in further studies.
